# Value and limitations of targeted next-generation sequencing in idiopathic hypereosinophilia: an integrative diagnostic tool in challenging cases

**DOI:** 10.1007/s10238-024-01441-w

**Published:** 2024-07-23

**Authors:** Daniele Cattaneo, Alfredo Marchetti, Cristina Bucelli, Nicole Galli, Marta Lionetti, Valentina Bellani, Umberto Gianelli, Francesco Passamonti, Niccolò Bolli, Alessandra Iurlo

**Affiliations:** 1 Hematology Division, Foundation IRCCS Ca’ Granda Ospedale Maggiore Policlinico, Milan, Italy; 2 Department of Oncology and Hemato-Oncology, University of Milan, Milan, Italy; 3 Division of Pathology, ASST Santi Paolo E Carlo, Milan, Italy; 4 Department of Health Sciences, University of Milan, Milan, Italy; 5 Hematology Division, Myeloproliferative Syndromes Unit, Foundation IRCCS Ca’ Granda Ospedale Maggiore Policlinico, Via Francesco Sforza 35, 20122 Milano, Italy

**Keywords:** Diagnosis, Hypereosinophilia, Mutations, Next-generation sequencing

## Abstract

Here, we reviewed clinical-morphological data and investigated mutational profiles by NGS in a single-center series of 28 consecutive patients admitted to our hospital between September 2011 and November 2021 for idiopathic hypereosinophilia (HE).

Bone marrow (BM) morphology was evaluated in 22 patients: while in six subjects BM was unremarkable, in the remaining cases an increase in BM eosinophils was observed, together with a slight increase in BM fibrosis (MF-1) in 5/22 patients.

A total of 4/28 patients had at least one genetic lesion by targeted NGS. In particular, the genes involved were: two each of *TET2* and *DNMT3A*; and one each of *JAK2*V617F, *ASXL1*, *PPM1D*, and *ZBTB33*. Notably, *JAK2*V617F and *TET2* mutations co-occurred, with the *JAK2*V617F-mutated sample also carrying *TET2* lesions. Median VAF was 21%, with the exception of the oncodriver *JAK2*V617F, which showed a VAF > 50% in the reported case. Of note, of the four cases bearing lesions, 2/4 had multiple hits in different genes.

While in recent years mutational analysis using NGS has proven to be able to differentiate clonal hematopoietic neoplasms from reactive processes in diagnostically difficult cases, we found somatic mutations in only 14.3% of patients who acceded to our hospital for idiopathic HE. More importantly, excluding the *JAK2*V617F-mutated case with an underlying MPN-Eo diagnosis, NGS was able to identify somatic mutations in only three cases, all older than 70 years. Consequently, the detection of these mutations in idiopathic HE patients should be interpreted with caution and only in the context of other supportive clinical-pathological findings.

## Introduction

Hypereosinophilic syndromes (HES) encompass a broad range of disorders characterized by persistent hypereosinophilia (HE) in the peripheral blood (PB) [i.e., an absolute eosinophil count (AEC) ≥ 1.5 × 10^9^/L and ≥ 10% eosinophils, preferably with a minimum duration of 6 months] associated with organ damage and/or dysfunction attributable to tissue eosinophilic infiltrate and release of granule contents. Its severity was arbitrarily divided into mild (AEC from the upper limit of normal to 1.5 × 10^9^/L), moderate (AEC 1.5–5 × 10^9^/L) and severe (AEC > 5 × 10^9^/L) [1].

Regarding hematological forms, the 2016 WHO Classification first approved a semi-molecular classification scheme of disease subtypes, including the following main categories: 1) myeloid/lymphoid neoplasms with eosinophilia (M/LN-Eo) and PDGFRA/B or FGFR1 rearrangement or with PCM1-JAK2; 2) myeloproliferative neoplasm (MPN) subtype: chronic eosinophilic leukemia, not otherwise specified (CEL, NOS); and 3) idiopathic HES (iHES), which is a diagnosis of exclusion [2].

More recently, the 2022 edition of the WHO Classification introduced several changes to the CEL diagnostic criteria, with the addition of the requirement for both clonality and abnormal bone marrow (BM) morphology [3]. In contrast, the contemporary ICC has refined the diagnostic criteria for iHES, emphasizing the importance of the absence of any molecular genetic clonal abnormality, with the caveat of clonal hematopoiesis (CH) of indeterminate potential [4, 5]. Indeed, the latter condition should always be taken into consideration when a pathogenic mutation, in particular involving the *ASXL1*, *DNMT3A*, or *TET2* genes, is detected with a low variant allele frequency (VAF) (e.g., ≤ 10%).

Therefore, once secondary causes of eosinophilia have been excluded, HES diagnostic work-up should be based on PB smear examination and blood tests (e.g., elevated serum B12 or tryptase level) in combination with BM morphological analysis, standard cytogenetic techniques, fluorescence in-situ hybridization, flow immunocytometry, evaluation of T-cell clonality, and molecular analysis [including next-generation sequencing (NGS) in selected cases] to detect histopathological or molecular evidence of acute or chronic myeloid or lymphoid neoplasms [6, 7].

In this study we aimed to molecularly characterize by NGS a single-center series of 28 consecutive patients admitted to our hospital between September 2011 and November 2021 for idiopathic HE.

## Material and methods

Inclusion criteria were as follows: available demographic and clinical-laboratory data at diagnosis; and at least one granulocyte DNA sample (collected upon first admission to our hospital for *JAK2*V617F mutation screening) to perform NGS analysis. All included cases had to meet the 2022 WHO and ICC minimum criteria for persistent HE [3–5], and underwent molecular analysis for *BCR::ABL1*, *TEL::PDGFRB*, *BCR::FGFR1* and *FIP1L1::PDGFRA* rearrangements by NESTED/RT-PCR. Cases of the so-called lymphocyte-variant HES, defined as a reactive condition secondary to immunophenotypically aberrant clonal T-cells, were excluded. Follow-up information was updated in October 2023.

NGS analysis was performed using DNA extracted from PB granulocytes. 100 ng of DNA was used to prepare libraries using the HyperPlus kit (KAPA Biosystems). The targeted gene panel included coding exons and splice sites of the following genes involved in myeloid malignancies based on literature data: CBLB, FBXW7, BRAF, RAD21, STAT5A, ATRX, ZBTB33, CSMD1, RB1, KIT, EZH2, KMT2D, ZNF318, EP300, GATA2, TP53, NLRP1, YLPM1, TET2, JAK2, SRCAP, ASXL1, FLT3, DDX18, NUP98, SETBP1, CSF3R, BCORL1, CSF1R, ZRSR2, CEBPA, SF3B1, CUX1, CREBBP, DNMT3A, PTPN11, SRSF2, JAK3, ABL1, NOTCH1, PPM1D, IDH2, CBL, NF1, STAT5B, STAG2, MECOM, KMT2A, MYC, WT1, NPM1, U2AF1. Variant interpretation was carried out as previously described [8–10]: in detail, libraries were sequenced using 150 bp paired end reads on Illumina MiSeq platform.

Mutect 2 GATK 4.3.0.0, LoFreq (v. 2.1.5), VarDict (2019.06.04), Freebayes (v. 1.3.6) and Strelka (v. 2.9.2) were used for variant calling, and only variants called by at least 2/5 of these variant callers were further considered. To filter out artifacts and germline polymorphisms, we first excluded variants with at least one of the following characteristics:—VAF lower than 0.02 and less than 15 supporting reads;—coverage < 300x;—occurrence in > 5% of a panel of normal controls (PoN, analyzed for the same gene panel and in the same sequencing conditions) at a VAF < 5%—expected minor germline allele frequency > 0.001 based on the information retrieved from the public database gnomAD (http://gnomad.broadinstitute.org; gnomAD r3.1.2);—or occurrence in at least one sample of our PoN at a VAF > 45% (both features being suggestive of polymorphic variants).

The shortlist of variants was therefore considered as containing bona fide somatic mutations, whose significance was evaluated at the amino acid level in order to differentiate known/putative pathogenic mutations from variants of unclear significance (VUS) on the basis of data available in literature and reported in the publicly accessible Catalogue Of Somatic Mutations In Cancer (COSMIC, version 69) (http://cancer.sanger.ac.uk/cancergenome/projects/cosmic).

Variants deemed as VUS were excluded from downstream analyses.

Median coverage was 1033 reads per base (IQR 791.5). Criteria for variant selection included a VAF greater than 2%, a Mean Allelic Frequency less than 1/1000 in the general population according to GnomAD [11], a coverage above 20, and an alternative allele count greater than 2. After variant selection, the median alternative allele count was 166 (IQR 117).

## Results

The clinical and laboratory features of the 28 patients included in the study are summarized in Table 1: after applying inclusion and exclusion criteria, they were diagnosed with CEL, NOS, myeloproliferative neoplasm with eosinophilia (MPN-Eo) or iHES in one patient each, or M/LN-Eo and PDGFRA rearrangement in four patients. Detailed characteristics of subjects diagnosed with underlying myeloid neoplasia are shown in Table 2. The remaining cases, due to the absence of organ damage by activated eosinophils (defined according to the proposal of the working group on eosinophilic disorders and syndromes) [1], were classified as HE of unknown significance.

**Table 1:** Clinical-laboratory features of the patients

	Patients n. 28
Male/female	18/10
Age at HE diagnosis, median (range)	54.7 (18.5–90.4)
AEC (× 10^9^/L), median (range)	2.4 (1.51–29.59)
Eosinophils (%), median (range)	28.5 (13.0–80.0)
WBC (× 10^9^/L), median (range)	9.3 (4.55–36.99)
Hb (g/dL), median (range)	13.6 (9.1–16.8)
PLT (× 10^9^/L), median (range)	248 (124–434)
LDH (IU/L), median (range)	198 (134–681)
Total IgE (kUA/L), median (range)	339 (6–1146)
Serum tryptase (mcg/L), median (range)	6 (3–15)
CRP (mg/dL), median (range)	0.21 (0.06–12.4)
Beta2microglobulin (mg/L), median (range)	2.1 (1.0–6.8)
Autoimmune screening positive, n (%)	
ANA	4 (14.3)
anti-dsDNA	1 (3.6)
ANCA	4 (14.3)
Splenomegaly, n (%)	3 (10.7)
Constitutional symptoms, n (%)	6 (21.4)
Organ damage, n (%)	1 (3.6)
Clinical presentation, n (%)	
Skin rashes/dermatitis	13 (46.4)
Pulmonary/Upper respiratory	8 (28.6)
Molecular screening, n (%)	
* JAK2*V617F mutation	1 (3.6)
* FIP1L1::PDGFRA* rearrangement	4 (14.3)
NGS, n (%)	
Wild-type	24 (85.7)
* TET2*	2 (7.1)
* DNMT3A*	2 (7.1)
* ASXL1*	1 (3.6)
* PPM1D*	1 (3.6)
* ZBTB33*	1 (3.6)
Bone marrow evaluation (N = 22), n (%)	
CEL, NOS	1 (4.5)
MPN-Eo	1 (4.5)
M/LN-Eo with PDGFRA rearrangement	4 (18.2)
Eosinophilic hyperplasia	10 (45.5)
Unremarkable	6 (27.3)
Bone marrow fibrosis (N = 22), n (%)	
MF-0	17 (77.3)
MF-1	5 (22.7)
Cytogenetic abnormalities (N = 22), n (%)	1 (4.5)
Follow-up from HE diagnosis (years), median (range)	3.9 (0.6–12.0)
Therapy, n (%)	
Steroids	9 (32.1)
Hydroxyurea	1 (3.6)
Imatinib	5 (17.9)

HE, hypereosinophilia; AEC, absolute eosinophil count; WBC, white blood cells; Hb, hemoglobin; PLT, platelets; LDH, lactate dehydrogenase; CRP, C-reactive protein; ANA, antinuclear antibody; ANCA, antineutrophil cytoplasmic antibody; CEL, NOS, chronic eosinophilic leukemia, not otherwise specified; MPN-Eo, myeloproliferative neoplasm with eosinophilia; M/L-Eo, myeloid/lymphoid neoplasm with eosinophilia

**Table 2:** Clinical-laboratory features of the patients with myeloid neoplasm-related hypereosinophilia

Case	Gender	Age at diagnosis (years)	AEC (× 10^9^/L)	WBC (× 10^9^/L)	Hb (g/dL)	PLT (× 10^9^/L)	LDH (IU/L)	Splenomegaly	Constitutional symptoms	Molecular screening	Diagnosis	Therapy	OS (years)
1	M	33.3	2.92	8.36	13.8	226	163	NO	YES	*FIP1L1::PDGFRA*	M/LN-Eo with PDGFRA rearrangement	Imatinib	5.2
2	M	43.1	17.69	23.59	13.9	252	217	NO	YES	*FIP1L1::PDGFRA*	M/LN-Eo with PDGFRA rearrangement	Imatinib	6.7
3	M	67.2	29.59	36.99	10.5	124	212	YES	NO	*FIP1L1::PDGFRA*	M/LN-Eo with PDGFRA rearrangement	Imatinib	2.5
4	M	33.2	6.45	12.49	14.8	162	180	YES	NO	*FIP1L1::PDGFRA*	M/LN-Eo with PDGFRA rearrangement	Imatinib	1.6
5	M	45.1	13.64	34.98	9.9	251	681	YES	YES	Negative	CEL, NOS	Imatinib	8.4
6	M	90.4	7.16	18.87	15.4	434	185	NO	NO	*JAK2*V617F	MPN-Eo	Hydroxyurea	5.3

AEC, absolute eosinophil count; WBC, white blood cells; Hb, hemoglobin; PLT, platelets; LDH, lactate dehydrogenase; M/L-Eo, myeloid/lymphoid neoplasm with eosinophilia; CEL, NOS, chronic eosinophilic leukemia, not otherwise specified; MPN-Eo, myeloproliferative neoplasm with eosinophilia; OS, overall survival

Clinically, patients most frequently presented with skin rashes and various forms of dermatitis (13/28, 46.4%), asthma or cough (8/28, 28.6%). Regarding the complete blood cell count, in addition to a median AEC of 2.4 × 10^9^/L, a higher-than-normal leukocyte count (defined as white blood cells ≥ 11 × 10^9^/L) was observed in 10/28 (35.7%) patients. Moderate anemia was reported in four (14.3%) cases, while platelet count was normal in all patients except one with mild thrombocytopenia. Interestingly, LDH, total serum IgE, and tryptase levels were higher than normal in nine (32.1%), 15 (53.6%), and 16 (57.1%) patients, respectively, while autoimmune tests were positive for ANA, anti-dsDNA or ANCA in nine (32.1%) patients in total, however without an underlying diagnosis of an autoimmune disease. Furthermore, parasitic diseases and allergies were excluded in all patients. Radiological examinations (chest X-ray) were unremarkable and electrocardiogram and echocardiogram showed good cardiac function in all cases, with the only exception of the patient diagnosed with iHES.

An abnormal karyotype (i.e., -Y) was observed in only one (4.5%) of 22 tested subjects. On the contrary, screening for *JAK2*V617F mutation was positive in one (3.6%) patients, while molecular analysis with NESTED/RT-PCR demonstrated the presence of the *FIP1L1::PDGFRA* rearrangement in four (14.3%) cases.

BM morphology was evaluated in 22 (78.6%) patients: while in six (27.3%) subjects BM was unremarkable, in the remaining cases an increase in BM eosinophils was observed, along with a slight increase in BM fibrosis (MF-1) in 5/22 (22.7%) patients. Importantly, ≥ 5% BM blasts and dysplasia in the megakaryocytic lineage were detected only in the patient diagnosed with CEL, NOS. As expected, the single case with *JAK2*V617F mutation showed overtly abnormal megakaryocytes, leading to a diagnosis of MPN-Eo.

**Fig. 1:**
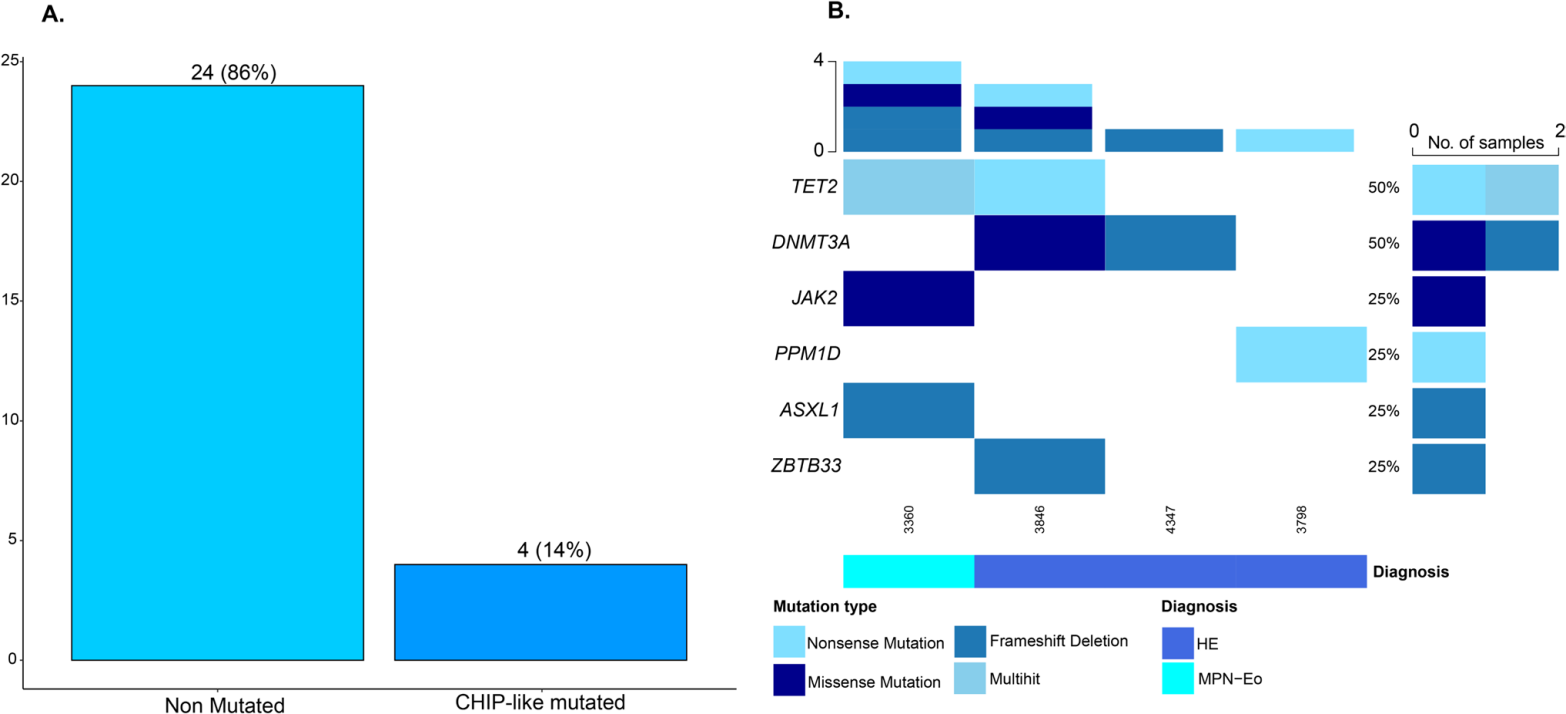
**A** Barplot expressing the number of samples without and with NGS-defined mutations. **B** Oncoplot depicting the mutational profile of the four mutated samples. Each column corresponds to a unique sample. The side bar plots the number of samples affected by mutations gene-wise, and the top side bar plots the mutation number per sample

28 samples underwent targeted NGS for recurrently mutated genes in myeloid neoplasms. Our pipeline, optimized for unmatched analysis, identifications of oncogenic and likely oncogenic variants and exclusion of germline polymorphisms and artifacts [8, 9], highlighted 8 gene mutations in 4 patients. A total of 4/28 (14.3%) patients had at least one relevant genetic lesion, with 2 patients bearing at least 2 mutations. The data and frequency of mutations are shown in Fig. 1. In particular, the genes involved were: two each (7.1%) of *TET2* and *DNMT3A*; and one each (3.6%) of *JAK2*V617F, *ASXL1*, *PPM1D*, and *ZBTB33*. Notably, *JAK2*V617F and *TET2* mutations co-occurred, with the *JAK2*V617F-mutated sample also carrying compound heterozygote *TET2* mutations. Median VAF was 21% (range, 3%—97%), with the exception of the oncodriver *JAK2*V617F, which showed a VAF > 50% in the reported case: in details, the VAF was 20% for *TET2*, 15% for *DNMT3A*, 42% for *ASXL1*, 6% for *PPM1D*, and 8% for *ZBTB33* mutation.

In details, all three *TET2* mutations in two patients were truncating, including nonsense stop codons p.Q1687* and pQ1501* or frameshift lesions as p.M1333fs. *DNMT3A* variants included one missense A368T and the frameshift G762fs. The single *PPM1D*, *ASXL1* and *ZBTB33* variants were represented by nonsense and frameshift lesions in the latter two cases: in particular, *PPM1D* variant was a nonsense lesion (amino acid change: p.C478*) involving exon 6 of the gene.

As expected, oral corticosteroids were the most frequently used drug in our series (9/28, 32.1%), except for the five patients with CEL, NOS or M/LN-Eo and PDGFRA rearrangement who were all treated with imatinib, achieving complete hematological remission; furthermore, hydroxyurea was used for cytoreduction in the case diagnosed with MPN-Eo.

After a median follow-up from the first admission to our hospital of 3.9 years (range, 0.6–12.0) only one death unrelated to HE (due to cognitive impairment) was recorded, while no new acute or chronic myeloid or lymphoid neoplasm was diagnosed in our patients’ population.

## Discussion

In recent years, NGS has contributed to identifying mutations in a large proportion of cases of myeloid neoplasms, including MPN, among which a consistent number of recurrent mutations appear to correlate with peculiar clinical features, prognosis, and treatment responses [12, 13]. Mutational analysis in general can help to differentiate a clonal hematopoietic neoplasm from a reactive process in diagnostically difficult cases. However, this approach has been complicated by reports of frequent somatic mutations in healthy populations of elderly individuals [14, 15].

The NGS panel used in this study included the most frequently mutated genes found in myeloid neoplasms, including genes encoding signaling molecules, transcription factors, epigenetic regulators, and splicing factors. Nevertheless, we found somatic mutations in only 14.3% of patients who acceded to our hospital due to idiopathic HE. The most frequent mutations found were in *TET2* and *DNMT3A* genes, both involved in DNA methylation; furthermore, except for the *JAK2*V617F-mutated case with MPN-Eo, other classic MPN driver mutations were not present in our patients’ series. Interestingly, no *KIT* mutations were found either, including *KIT*M541L which was associated with an optimal response to imatinib treatment in four of five CEL, NOS patients who did not have PDGFRA/B lesions [16].

Focusing specifically on the four patients with M/LN-Eo and PDGFRA rearrangement, we did not observe any frequent or recurrent cooperating mutations, including in known cancer genes or in genes associated with age-related CH, thus confirming their distinct molecular pathogenesis [17], responsible for a robust and sustained response to tyrosine kinase inhibitors [18].

More importantly, the *JAK2*V617F-mutated case with an underlying MPN-Eo diagnosis carried 4 out of 9 mutations identified in the whole cohort, thus showing a clearly different mutational pattern. Conversely, NGS was able to identify at least one somatic mutation in only three (10.7%) additional cases, all older than 70 years, thus with a prevalence similar to the general population. Consequently, assuming that somatic mutations in genes associated with myeloid neoplasms (such as *DNMT3A*, *TET2*, *ASXL1*, *JAK2*, *TP53*, *PPM1D*, *GNAS*, *BCORL1*, and *SF3B1*) have also been frequently found in healthy aging individuals [14, 15, 19], and that, although individuals with acquired somatic mutations may have an increased risk of developing a myeloid neoplasm such as myelodysplastic syndrome or acute myeloid leukemia [20], the complex dynamics of CH are highly variable and are not necessarily predictable [21], the detection of these mutations in patients with idiopathic HE and their putative role in the pathogenetic mechanisms of these conditions should be interpreted with caution and only in the context of other supportive clinical and pathological findings.

Therefore, although the classification of myeloid neoplasms with eosinophilia is increasingly based on molecular markers [22, 23], and, with the widespread availability of NGS panels, the identification of additional mutations in cases of idiopathic HE/HES is expected to be more common, with an increasing number of patients potentially assigned to the CEL, NOS category, a diagnosis of clonal HE should still be anchored on a combination of histomorphological, clinical-laboratory and molecular criteria.

## Data Availability

The data that support the findings of this study are available from the authors upon reasonable request.
